# Associations Between Restoration Margins and Adjacent Periodontal Status—Longitudinal Results From SHIP‐TREND

**DOI:** 10.1111/jcpe.70082

**Published:** 2025-12-22

**Authors:** Patrick Nafz, Thomas Kocher, Christiane Pink, Sebastian‐Edgar Baumeister, Stefan Reckelkamm, Stefanie Samietz, Sonya Nafz, Henry Völzke, Philipp Kanzow, Birte Holtfreter

**Affiliations:** ^1^ Department of Restorative Dentistry, Periodontology and Endodontology University Medicine Greifswald Greifswald Germany; ^2^ Institute of Health Services Research in Dentistry, University of Münster Münster Germany; ^3^ Clinic for Periodontology and Conservative Dentistry, University of Münster Münster Germany; ^4^ Department of Prosthodontics, Gerodontology and Dental Materials University Medicine Greifswald Greifswald Germany; ^5^ Institute for Community Medicine, SHIP/Clinical‐Epidemiological Research, University Medicine Greifswald Greifswald Germany; ^6^ German Centre for Cardiovascular Research (DZHK), Partner Site Greifswald Greifswald Germany

**Keywords:** cohort study, epidemiology, periodontal health, restoration margin, restoration status, risk factor

## Abstract

**Aim:**

To investigate the association between dental restorations and adjacent periodontal status over a 7‐year period, using data from a population‐based cohort study.

**Materials and Methods:**

We used 7‐year follow‐up data on the restorative and periodontal statuses of 88,793 tooth surfaces from 2158 SHIP‐TREND (Study of Health in Pomerania) participants. Using confounder‐adjusted and inverse‐probability‐weighted generalised estimating equations, we estimated the associations of restoration status with bleeding on probing (BOP), probing depth (PD) and clinical attachment level (CAL).

**Results:**

Surfaces with dental restorations had significantly poorer periodontal outcomes than sound surfaces, with crowns having the greatest impact. At follow‐up, filled and crowned surfaces presented higher proportions of adjacent sites with BOP (18.5% and 22.4%, respectively) compared to sound surfaces (15.8%). Similarly, adjusted average PD was 1.93 mm adjacent to sound surfaces, 1.99 mm adjacent to surfaces with fillings and 2.14 mm adjacent to surfaces with crowns. The results remained consistent when the effects of incidentally placed fillings and crowns on follow‐up periodontal status were evaluated. Although effect modification by surface type was observed, no consistent patterns emerged across the different outcomes.

**Conclusion:**

Dental restorations can have an adverse effect on periodontal health, emphasising the critical need for precise restorative techniques and post‐treatment maintenance.

## 1 Introduction

Periodontitis is an inflammatory disease affecting the supporting tissue of the teeth (Chapple et al. 2018; Murakami et al. 2018; Papapanou et al. 2018). Alongside dental caries, it ranks among the most common oral diseases globally (Trindade et al. 2023). In 2021, over 1 billion people worldwide suffered from severe periodontitis, a number projected to rise significantly in the coming decades due to ageing populations and population growth (Nascimento et al. 2024). Improved restorative care has resulted in increased tooth retention in older populations, which increases the number of teeth potentially requiring periodontal treatment.

Restorations have been consistently associated with adverse periodontal outcomes (Giollo et al. 2007; Jansson et al. 1994; Pack et al. 1990; Pitchika et al. 2026; Schatzle et al. 2001). An early study of amalgam restorations found that periodontal pockets adjacent to restoration margins were nearly 2–3 (depending on the quality of the restoration) times more likely to exceed 3 mm in depth than sound surfaces (Pack et al. 1990). Similar differences were observed for bleeding on probing (BOP), with bleeding occurring almost 3 times more frequently at sites adjacent to restorations compared with sound surfaces (Pack et al. 1990). Two more recent cross‐sectional studies found that the mean clinical attachment levels (CALs) were 0.13 mm (Giollo et al. 2007) and 0.22–0.37 mm (depending on age group and measurement site) higher for crowned teeth than for sound teeth (Pitchika et al. 2026). These findings raise the question of whether the relationship between restorations and periodontal health has changed over time. Considering the substantial advances in restorative materials and techniques in recent decades, well‐designed longitudinal studies are needed to re‐evaluate this association in the context of modern clinical practice.

Therefore, we examined the impact of existing restorations (distinguishing between fillings and partial/full crowns) and newly placed restorations on periodontal health at follow‐up using data from the longitudinal Study of Health in Pomerania (SHIP‐TREND). Our findings provide valuable insights into the complex interplay between restorative dental treatments and periodontal health.

## 2 Material and Methods

### 2.1 Study Design

SHIP‐TREND is a population‐based observational study conducted in northeast Germany (Völzke et al. 2022). A stratified random sample of 10,000 adults aged 20–79 years was drawn from population registries. Stratification variables were age, sex and city/county of residence. Migrants (*N* = 851) and deceased persons (*N* = 323) were excluded from the random sample of 10,000 adults, leaving 8826 persons in the net sample. Of these, 4420 were recruited for the baseline study, yielding a response of 50.1%. Baseline examinations took place between 2008 and 2012 (SHIP‐TREND‐0). Follow‐up examinations were conducted between 2014 and 2018 (SHIP‐TREND‐1) with 2507 participants. Follow‐up periods ranged from 4.9 to 10.3 years, with an average of 7.4 years. Figure S1 illustrates the data selection process for our analysis. Records with missing follow‐up data, baseline restoration status or confounder information were excluded, leaving 96,984 surfaces for final analysis.

### 2.2 Dental Examinations and Exposure Definition

Detailed information on caries and periodontal examinations is provided in the Supporting Information. Restoration status (exposure) was defined as ‘sound’, filled or filled with secondary caries (referred to as ‘filled’) or full or partial crown (referred to as ‘crowned’). Surfaces with primary caries were excluded from analyses. For surface‐/site‐level analyses, periodontal sites were allocated to tooth surfaces (restoration status), with sites and surfaces allocated to each other based on proximity: disto‐buccal site with distal surface, mid‐buccal site with buccal surface, mesio‐buccal site with mesial surface and mid‐palatinal/mid‐lingual site with palatinal/lingual surface.

Detailed information on laboratory measurements and covariates and reliability data are provided in Supporting Information.

### 2.3 Statistical Analyses

Means and standard deviations (SD), as well as medians with 25% and 75% quantiles were reported for continuous variables. Relative frequency distributions were computed for categorical variables.

The association between baseline restoration status and periodontal outcomes at the site/surface level at follow‐up was modelled using generalised estimating equations with cluster‐robust standard errors to estimate population‐averaged estimates (Model A; newly placed or replaced restorations were not considered). Modified Poisson regression was used for the presence of BOP and the presence of PD ≥ 4 mm, while gamma distribution and a log link were used for PD and CAL. All models were weighted using inverse probability weights to account for differential loss to follow‐up (the model that predicted follow‐up participation included sex, school education, income, number of medications and physician visits, hypertension, smoking status, cancer diagnosis and stroke diagnosis; missing values of predictors were imputed; weights were trimmed at the 99% percentile; the area under the curve was 0.735). Confounder‐adjusted effect estimates were presented as risk ratios (RRs) with corresponding 95% confidence intervals (CI) or as exp(β) with corresponding 95% CIs and interpreted as a percent change of the outcome (Manning et al. 2005). Confounders were chosen according to the modified disjunctive cause criterion (VanderWeele 2019). Accordingly, we adjusted models for baseline levels of the periodontitis variable, age (cont.), sex, school education, smoking, known or diagnosed diabetes mellitus, body mass index (cont.), dental visit within the last 12 months, probing site and the food frequency score (FFS) pattern. Non‐linear forms of continuous variables did not benefit model performance compared to linear forms. For the calculation of marginal means, the following settings were fixed: baseline BOP = 0; baseline PD = 2, baseline PD ≥ 4mm = 0, baseline CAL = 2, age = 50, sex = female, school education = 10 years, smoking = never, diabetes mellitus = no, BMI = 27, dental visit = no, probing site = mid‐oral and FFS pattern = 14. Missing covariate data were not imputed because less than 5% of covariates were missing. We also provided models additionally adjusted for household equivalence income (see Table S1; for calculation of marginal means, household equivalence income was set to €1500).

We evaluated an effect modification by surface type on the outcome and included a multiplicative interaction term between surface (proximal vs. non‐proximal) and the exposure variable in regression models (*p* for interaction < 0.10). As most of the interaction terms were significant, we evaluated the models separately for the proximal (distal and mesial surfaces; for calculation of marginal means, probing site was set to ‘distal’) and the non‐proximal surfaces (mid‐oral and buccal surfaces; for calculation of marginal means, probing site was set to ‘mid‐oral’).

Finally, we calculated incidence rates per 1000 person‐years stratified by baseline restoration status. The worst restoration status per tooth was defined considering oral, buccal, distal, mesial and occlusal surfaces. Analyses included 25,714 teeth in 2276 subjects with baseline caries data and follow‐up information on tooth loss. For the extracted teeth, the event time was set at half the difference between the baseline and the follow‐up.

A two‐sided *p* < 0.05 was considered statistically significant. All analyses were performed using Stata/SE version 17.0 (StataCorp 2021) and R 4.3.3 (R Core Team 2024).

## 3 Results

### 3.1 Baseline Characteristics

Table 1 shows the baseline characteristics, highlighting key demographic and behavioural factors. The average age was 48.7 years (SD = 13.5) and 48.3% were male. The majority (55.7%) completed 10 years of schooling, while 32.8% had completed more than 10 years. Twenty‐three percent were current smokers, and 30% used powered toothbrushes alone or in combination with manual ones. Notably, 29.3% of participants reported using interdental cleaning aids, and 92% reported visiting a dentist within the past year.

**TABLE 1:** Baseline characteristics (for subjects included in any of the models from Table 3; *N* = 2169).

	Total
Age, years	48.7 ± 13.5 48 (38, 59)
Male sex	1048 (48.3%)
School education	
< 10 years	250 (11.5%)
10 years	1207 (55.7%)
> 10 years	712 (32.8%)
Household equivalence income (€)	1503.0 ± 741.4 1449.6 (1096.0, 1803.1)
Missing	73
Smoking status	
Never smoker	844 (38.9%)
Former smoker	828 (38.2%)
Current smoker	497 (22.9%)
Body mass index, kg/m^2^	27.4 ± 4.6 27.0 (24.1, 30.0)
Known or diagnosed diabetes, yes	156 (7.2%)
Food frequency score	13.9 ± 3.4 14 (11, 16)
Toothbrush use	
No toothbrush use	8 (0.4%)
MTB use	1521 (70.1)
Both MTB and PTB use	152 (7.0%)
PTB use	488 (22.5%)
Interdental care, yes	636 (29.3%)
Toothbrushing frequency	
< 2 times daily	273 (12.6%)
≥ 2 times daily	1896 (87.4%)
Dental visit within the last 12 months, yes	1995 (92.0%)
Self‐reported periodontal treatment within last 5 years, yes	432 (20.0%)
Regular professional tooth cleaning (≥ 1 times/year), yes	682 (31.5%)

*Note:* Data are presented as mean ± standard deviation and median (Q25%, Q75%) or numbers (percentages).

Abbreviations: MTB, manual toothbrush; PTB, powered toothbrush.

### 3.2 Observed Changes in Periodontal Status According to Caries/Crown Status

**FIGURE 1:**
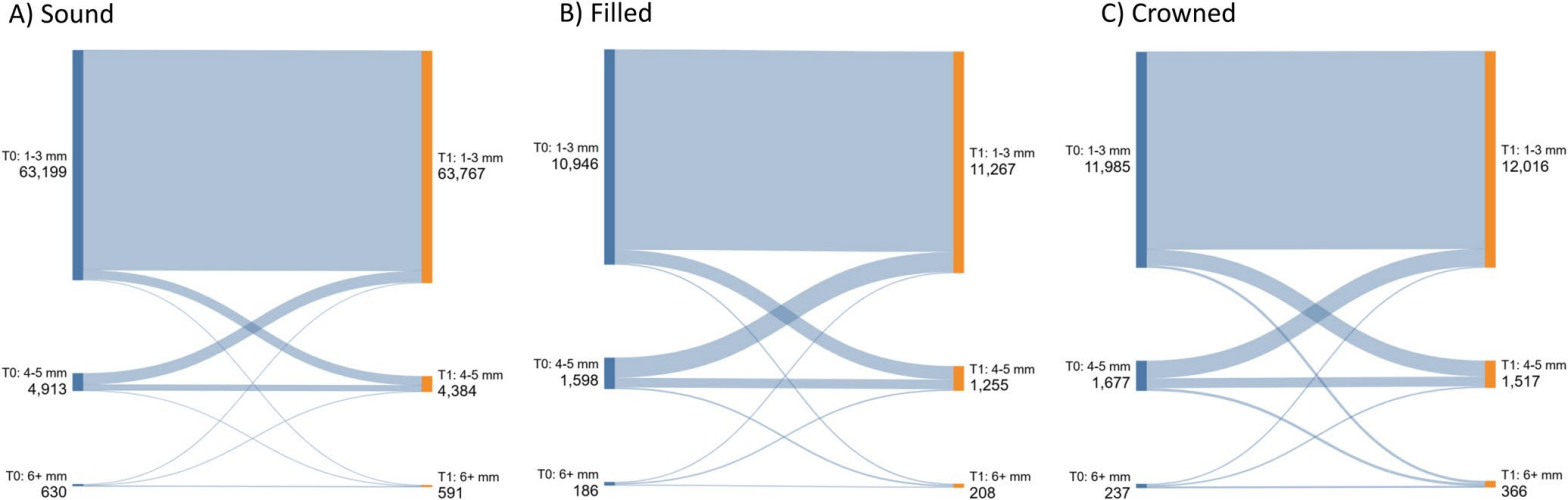
Sankey plots showing shifts in probing depths between baseline (SHIP‐TREND‐0; T0) and 7‐year follow‐up (SHIP‐TREND‐1; T1) by baseline restoration status.

Table 2 shows the changes in the observed periodontal parameters between baseline and follow‐up. The percentage of surfaces with BOP decreased slightly from 21.1% at baseline to 17.9% at follow‐up. PD was marginally reduced from 2.41 ± 0.96 mm at baseline to 2.34 ± 0.97 mm at follow‐up. Meanwhile, CAL showed a slight increase, shifting from 1.84 ± 1.56 mm at baseline to 2.01 ± 1.50 mm at follow‐up. Graphical analysis of PD transitions between baseline and 7‐year follow‐up (Figure 1) revealed shifts from 1–3 to 4–5 mm and from 4–5 to 1–3 mm across all groups. However, for the filled and crowned group, the shift towards more severe PDs was only slightly more pronounced compared to the other two groups.

**TABLE 2:** Baseline and 7‐year follow‐up surface‐level data in total and by restoration status.

				By baseline restoration status					
		Total		Sound		Filled		Crowned	
		*N*	*N* pos (%) or mean ± SD and median (Q25%, Q75%)	*N*	*N* pos (%) or mean ± SD and median (Q25%, Q75%)	*N*	*N* pos (%) or mean ± SD and median (Q25%, Q75%)	*N*	*N* pos (%) or mean ± SD and median (Q25%, Q75%)
BOP, yes	Baseline	44,901	9487 (21.1%)	31,437	6047 (19.2%)	6105	1393 (22.8%)	7359	2047 (27.8%)
	7‐year follow‐up	44,901	8041 (17.9%)	31,437	5000 (15.9%)	6105	1221 (20.0%)	7359	1820 (24.7%)
PD, mm	Baseline	95,371	2.41 ± 0.96 2 (2, 3)	68,742	2.32 ± 0.93 2 (2, 3)	12,730	2.63 ± 0.98 2 (2, 3)	13,899	2.65 ± 0.99 2 (2, 3)
	7‐year follow‐up	95,371	2.34 ± 0.97 2 (2, 3)	68,742	2.26 ± 0.91 2 (2, 3)	12,730	2.50 ± 1.00 2 (2, 3)	13,899	2.59 ± 1.14 2 (2, 3)
PD ≥ 4 mm, yes	Baseline	95,371	9241 (9.7%)	68,742	5543 (8.1%)	12,730	1784 (14.0%)	13,899	1914 (13.8%)
	7‐year follow‐up	95,371	8321 (8.7%)	68,742	4975 (7.2%)	12,730	1463 (11.5%)	13,899	1883 (13.6%)
CAL, mm	Baseline	77,035	1.84 ± 1.56 2 (1, 3)	65,708	1.80 ± 1.55 2 (1, 3)	11,029	2.10 ± 1.62 2 (1, 3)	298	2.20 ± 1.48 2 (1, 3)
	7‐year follow‐up	77,035	2.01 ± 1.50 2 (1, 2)	65,708	1.97 ± 1.49 2 (1, 2)	11,029	2.20 ± 1.57 2 (1, 3)	298	2.28 ± 1.42 2 (1, 3)

*Note:* BOP was measured only at the first incisors, canines and first molars.

Abbreviations: BOP, bleeding on probing; CAL, clinical attachment level; *N* pos, number of positive cases; PD, probing depth; SD, standard deviation.

### 3.3 Associations of Caries/Crown Status With Periodontal Status

Two models were set up to estimate associations of restoration status with periodontal status at follow‐up (Table 3). Overall, the presence of fillings and crown margins was consistently associated with worse periodontal status at follow‐up.

**TABLE 3:** Adjusted effects estimates from inverse‐probability‐weighted generalised estimating equations with cluster‐robust standard errors (BOP and PD ≥ 4 mm: Modified Poisson regression; PD, CAL: Gamma distribution and log link; with inverse probability weighting and robust standard errors) obtained by regressing the periodontal status at follow‐up in Model A on baseline caries status (both from partial‐mouth examinations) and in Model B on the change in caries status between baseline and 7‐year follow‐up (incident fillings and crowns).

	Outcome			
	Having bleeding on probing	Probing depth	Having a probing depth of ≥ 4 mm	Clinical attachment level
Model A	*N* = 44,901	*N* = 95,371	*N* = 95,371	*N* = 77,035
Number (%) of surfaces being				
Sound	31,437 (70.0%)	68,742 (72.1%)	68,742 (72.1%)	65,708 (85.3%)
Filled	6105 (13.6%)	12,730 (13.3%)	12,730 (13.3%)	11,029 (14.3%)
Crowned	7359 (16.4%)	13,899 (14.6%)	13,899 (14.6%)	298 (0.4%)
Effect estimates (with 95% CIs)	RR with 95% CI	Exp(β) with 95% CI	RR with 95% CI	Exp(β) with 95% CI
Sound	Ref. (1.00)	Ref. (1.00)	Ref. (1.00)	Ref. (1.00)
Filled	1.17 (1.10–1.24)	1.03 (1.02–1.04)	1.18 (1.11–1.24)	1.04 (1.03–1.05)
Crowned	1.41 (1.33–1.50)	1.11 (1.09–1.12)	1.47 (1.38–1.56)	1.20 (1.11–1.29)
Adjusted percentages/averages (with 95% CIs)				
Sound	15.8 (13.3–18.4)	1.93 (1.87–1.99)	4.6 (3.6–5.6)	1.71 (1.59–1.82)
Filled	18.5 (15.4–21.6)	1.99 (1.93–2.05)	5.4 (4.2–6.6)	1.78 (1.66–1.90)
Crowned	22.4 (18.7–26.0)	2.14 (2.07–2.20)	6.7 (5.2–8.2)	2.05 (1.84–2.25)
Model B	*N* = 31,360	*N* = 68,593	*N* = 68,593	*N* = 65,618
Number (%) of surfaces being				
Sound	28,993 (92.5%)	63,470 (92.5%)	63,470 (92.5%)	62,618 (95.4%)
Incidentally filled	1443 (4.6%)	2989 (4.4%)	2989 (4.4%)	2910 (4.4%)
Incidentally crowned	924 (2.9%)	2134 (3.1%)	2134 (3.1%)	90 (0.1%)
Effect estimates (with 95% CIs)	RR with 95% CI	Exp(β) with 95% CI	RR with 95% CI	Exp(β) with 95% CI
Sound	Ref. (1.00)	Ref. (1.00)	Ref. (1.00)	Ref. (1.00)
Incidentally filled	1.23 (1.11–1.37)	1.03 (1.02–1.05)	1.15 (1.04–1.27)	1.02 (1.002–1.04)
Incidentally crowned	1.38 (1.21–1.57)	1.09 (1.07–1.12)	1.27 (1.10–1.48)	1.14 (1.03–1.27)
Adjusted percentages/averages (with 95% CIs)				
Sound	16.0 (12.9–19.0)	1.90 (1.84–1.96)	3.7 (2.8–4.6)	1.73 (1.61–1.84)
Incidentally filled	19.7 (15.5–23.9)	1.97 (1.90–2.03)	4.2 (3.1–5.4)	1.76 (1.64–1.89)
Incidentally crowned	22.0 (17.1–26.8)	2.08 (2.00–2.16)	4.7 (3.3–6.0)	1.97 (1.72–2.22)

*Note:* For Model B, we included only sites that were sound and caries‐free at baseline. Models were adjusted for baseline levels of the outcome variable, probing site, age (cont.), sex, education, smoking, known or diagnosed diabetes, body mass index (cont.), dental visit within last 12 months and food frequency score (cont.). The following settings were fixed for the calculation of marginal means: baseline BOP = no; baseline PD = 2, baseline PD ≥ 4 mm = no, baseline CAL = 2, age = 50, sex = female, school education = 10 years, smoking = never, diabetes mellitus = no, BMI = 27, dental visit = no, probing site = mid‐oral and FFS = 14. BOP was only measured at the first incisors, canines and first molars.

Abbreviations: CI, confidence interval; RR, risk ratio; β, beta coefficient.

**FIGURE 2:**
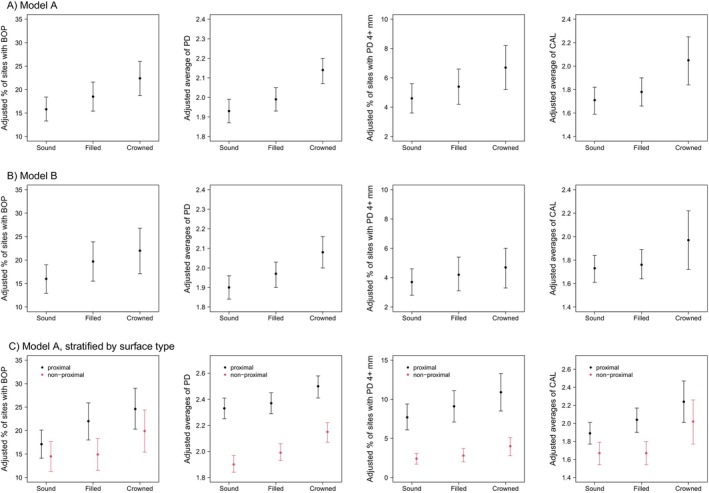
Adjusted follow‐up levels of the percentage of sites with bleeding on probing (BOP), average probing depth (PD), percentage of sites with PD ≥ 4 mm and average clinical attachment level (CAL) for (A) model A, (B) model B (for both models see Table 3) and (C) model A stratified by surface type (for models see Table S2). BOP was measured only at the first incisors, canines and first molars.

Model A estimated the adjusted fixed effects of baseline restoration status on follow‐up periodontal status. Compared to sound surfaces, filled surfaces showed a 1.17‐fold higher risk of BOP at follow‐up (95% CI: 1.10–1.24), while crowned surfaces showed a 1.41‐fold higher risk (95% CI: 1.33–1.50). The adjusted percentage of sites with BOP was 15.8% for sound surfaces, 18.5% for filled surfaces and 22.4% for crowned surfaces (see also Figure 2, upper panel). PD increased by 3% (exp(β) = 1.03; 95% CI: 1.02–1.04) for filled surfaces and by 11% (exp(β) = 1.11; 95% CI: 1.09–1.12) for crowned surfaces compared to sound surfaces. Accordingly, the adjusted average PD values were 1.93, 1.99 and 2.14 mm. Compared to sound surfaces, filled surfaces showed a 1.18‐fold (95% CI: 1.11–1.24) higher risk of having PD ≥ 4 mm at follow‐up, while for crowned surfaces the risk was 1.47‐fold higher (95% CI: 1.38–1.56). The adjusted percentage of sites with PD ≥ 4 mm was 4.6%, 5.4% and 6.7%. Similarly, the adjusted average CALs were 1.71, 1.78 and 2.05 mm for sound, filled and crowned surfaces, respectively.

Model B assessed the association between changes in restoration status (limited to surfaces that were sound at baseline; Table 3) and periodontal status at follow‐up, assuming that restorations preceded changes in periodontal status. Specifically, the risk for BOP was 1.23‐fold (95% CI: 1.11–1.37) higher for incidentally filled surfaces and 1.38‐fold (95% CI: 1.21–1.57) higher for incidentally crowned surfaces compared to sound surfaces. The adjusted percentage of sites with BOP was 16.0%, 19.7% and 22.0%, respectively (Figure 2, middle panel). The adjusted average PD values for sound, incidentally filled, and incidentally crowned surfaces were 1.90, 1.97 and 2.08 mm, respectively. The adjusted percentage of sites with PD ≥ 4 mm was 3.7%, 4.2% and 4.7%. Correspondingly, the adjusted average CAL values were 1.73 mm for sound surfaces, 1.76 mm for incidentally filled surfaces and 1.97 mm for incidentally crowned surfaces. Additional adjustment of Models A and B for household equivalence income did not change effect estimates materially (see Table S1).

### 3.4 Effect Modification by Surface Type

Finally, we evaluated effect modification by surface type (see Table S2 and Figure 2, lower panel). However, no consistent pattern emerged. More pronounced effects were found for proximal surfaces in BOP and CAL (specifically for crowns), whereas PD models showed more pronounced effects for non‐proximal surfaces. Periodontal parameters were consistently higher for proximal surfaces than for non‐proximal ones.

### 3.5 Extraction Patterns According to Restoration Status

We additionally evaluated the incidence rates for extraction according to worst restoration status (considering all surfaces per tooth) on tooth level (see Table S3). Incidence rates were significantly higher for filled (7.4 per 1000 person‐years; 95% CI: 6.7–8.2) and crowned teeth (17.7 per 1000 person‐years; 95% CI: 16.2–19.3) compared to sound teeth (2.8 per 1000 person‐years, 95% CI: 2.5–3.2).

## 4 Discussion

In the context of limited longitudinal evidence, this study provides new insights into the impact of dental restorations on periodontal health. Apart from the Dunedin study, no other population‐based cohort study has addressed this relationship comprehensively. Our results demonstrated significantly poorer periodontal parameters at sites adjacent to all types of restorations than at sound surfaces, with the most pronounced detrimental effects adjacent to full or partial crown margins. Our data show that deterioration is already evident by the first follow‐up. Considering known chronic periodontitis progression rates (0.07–0.28 mm/year) (Gatke et al. 2012; Papapanou et al. 1989), this reflects early but expected changes rather than unusually rapid progression. Nonetheless, it highlights that clinically relevant deterioration can occur sooner than often anticipated, supporting close monitoring shortly after prosthetic placement. At follow‐up, adjusted averages of BOP, PD and CAL at sites adjacent to incidentally placed restorations were comparable to those at sites adjacent to older restorations (Table 3, Model A). Thus, our data suggest that adverse periodontal changes may occur rapidly after prosthetic placement and stabilise shortly afterwards, therefore supporting the theory of re‐establishment of the biological width.

Previous research has demonstrated that the quality of dental restorations, particularly the marginal fit of crowns and fillings, might significantly impact periodontal tissues (Brunsvold and Lane 1990; Gilmore and Sheiham 1971; Jeffcoat and Howell 1980; Lang et al. 1983; Schatzle et al. 2001). Overhanging margins have consistently been associated with periodontal deterioration: (i) A significantly higher severity of periodontal disease at sites adjacent to dental restorations was observed, affecting 20%–30% of approximal fillings, depending on the patient's age (Gilmore and Sheiham 1971); (ii) Increasing gingival indices were detected at sites with overhanging margins (Lang et al. 1983); (iii) Greater bone loss was found around teeth with overhanging restorations (Jeffcoat and Howell 1980). Furthermore, restorations placed below the gingival margin were detrimental to gingival and periodontal health (Schatzle et al. 2001).

Most existing studies on this topic were published before 2000, resulting in a lack of recent research. Since then, significant changes have been observed in the prevalence of overhanging margins, suggesting an improvement in restoration quality over time. For instance, an early study found that 62% of all approximal fillings had overhanging margins (Pack et al. 1990). By 2009, this figure had fallen to 14.1% (Kuonen et al. 2009). The most recent study, the Dunedin study, found that surfaces with fillings were almost twice as likely to have a PD of ≥ 3 mm as intact surfaces (Broadbent et al. 2006). The most recently published cross‐sectional study (Pitchika et al. 2026) found that PD values were about 0.16 (0.27) and 0.22 (0.37) mm higher at sites adjacent to fillings and crowns than at healthy surfaces in individuals aged 35–44 (65–74) years. CAL was 0.27 and 0.37 mm higher at sites adjacent to fillings than at healthy surfaces in both age groups. Unlike older studies—but in line with that of Pitchika et al. (2026)—our study found much smaller effects, which may reflect the generally higher quality of restorations used today, particularly in high‐income countries. As in previous studies, we observed that periodontal outcomes were poorer adjacent to restorations, especially crowns. However, as 63% of crowns are placed subgingivally, one might question whether the higher periodontal parameters are primarily attributable to subgingival margin placement (Giollo et al. 2007).

The deterioration in periodontal health observed in association with restorative treatments may be attributed to a combination of mechanical and biological factors. Restoration margins can facilitate biofilm accumulation, provoke gingival irritation and trigger an inflammatory response that can progress to gingivitis, a recognised precursor to periodontitis (Lang et al. 2009). At the microbiome level, sites adjacent to crowns exhibited a higher, albeit not statistically significant, presence of putative periodontopathogens, including *Porphyromonas gingivalis, Treponema denticola* and *Tannerella forsythia* (Giollo et al. 2007). The biological width (Gargiulo et al. 1961; Vacek et al. 1994) plays a crucial role in periodontal outcomes, as subgingival restorations can alter the highly diverse microbiome (Mira et al. 2017) at the gingival margin, thereby disrupting the epithelial barrier and connective tissue attachment. This disruption may result in increased BOP, as well as increased levels of PD and CAL.

Owing to challenges such as poorer oral hygiene and the increased treatment complexity in proximal areas, we suspected an effect modification by the surface type. Although such modification was statistically present, it was inconsistent across periodontal outcomes (see Figure 2, lower panel, and Table S2), with more pronounced effects for filled or crowned surfaces being observed for proximal surfaces or non‐proximal surfaces, depending on the periodontal outcome. In line with the literature (Carvalho et al. 2023; Farina et al. 2011; Sager et al. 2023), periodontal disease levels were worse at proximal sites than at non‐proximal sites. However, there were no clinically relevant differences in the adjusted effects of fillings and crowns on periodontal outcomes.

The following strengths of our study are worth noting. First, this is the first large‐scale cohort study in over two decades to examine the relationship between dental restorations and periodontal health using 7‐year follow‐up data. It provides robust insights into the mid‐term effects of fillings and crowns on periodontal outcomes. The use of three confounder‐adjusted regression models with different model set‐ups strengthens the validity of our findings. A second key strength is the high level of examiner expertise, with trained dentists undergoing regular calibration to ensure consistent and reliable periodontal measurements.

However, some limitations must also be mentioned. First, as SHIP‐TREND participants are Caucasian, the results may not be fully generalisable to other populations with different genetic backgrounds, access to health care and oral hygiene practices. Second, a key limitation of this study is the response rate of approximately 50% in the SHIP‐TREND‐1 cohort, which may introduce selection and attrition bias. Healthier individuals were more likely to participate, leading to a selection bias that could affect the generalisability of the findings. For example, individuals with more severe periodontal conditions might be underrepresented. This may result in an underestimation of the true effect sizes. Third, some limitations were connected to the periodontal protocol: (i) Periodontal status was assessed using half‐mouth protocols, with only four sites measured instead of six; (ii) BOP was only assessed at the first incisors, canines and first molars; (iii) Proximal sites comprised only the mesio‐buccal and disto‐buccal sites, and non‐proximal sites comprised the mid‐buccal site only. Although it can be assumed that the effect estimates are similar regardless of the site examined, the composition of the proximal and non‐proximal sites may have affected the estimates to an unknown extent and direction. This could have led to the associations with restoration status being underestimated. Fourth, although we have made fine adjustments for various confounders, factors such as genetic predisposition leave the potential for residual confounding. Fifth, no information was available regarding the exact timing of dental restoration placement during the follow‐up period. Therefore, some degree of periodontal deterioration might have already occurred before the restoration was placed, potentially leading to an overestimation of restoration‐associated periodontal effects. Sixth, CAL measurements were omitted when the cemento‐enamel junction (CEJ) was obscured by (large) restorations or crown margins (likely associated with subgingival margins). Thus, only a few sites with available CAL measurements, mainly from partial crowns, were available for analysis. Therefore, CAL results should be interpreted with caution. Finally, a major limitation of this study is that we did not assess the quality, material or technical characteristics of the restorations, including the presence of overhanging margins or subgingival placement. Previous research has shown that these factors are strongly associated with increased gingival inflammation, greater bone loss and more severe periodontal disease adjacent to restorations (Gilmore and Sheiham 1971; Jeffcoat and Howell 1980; Pack et al. 1990). Our study could not account for these important contributors.

Advances in restorative dentistry and periodontal management have significant potential to improve long‐term outcomes. Future research should focus on developing restorative techniques that minimise overhangs and subgingival placement, as numerous studies have identified these factors as key contributors to periodontal deterioration and cleaning of the approximate surfaces (Brunsvold and Lane 1990; Gilmore and Sheiham 1971; Jeffcoat and Howell 1980; Lang et al. 1983; Pack et al. 1990).

Considering the worse periodontal outcomes observed around crowns compared to fillings, future research should explore whether extending the lifespan of fillings, restricting indications for crown placement, the predominance of subgingival margins or specific crown materials contributes to the observed effects on periodontal health.

From a clinical perspective, it should be noted that, while the proportion of surfaces with PD ≥ 4 mm (indicating the need for periodontal treatment) increased from 4.6% at sound surfaces to 6.7% at crowned surfaces (Table 3), the proportion of sites with BOP was 7% higher at crowned surfaces than at sound surfaces. Therefore, in the short term, increased gingivitis treatment needs are more clinically relevant than intensive periodontal treatment needs. However, to prevent the progression of gingivitis to chronic periodontitis in the long term, this should not be ignored. In summary, these findings emphasise the importance of precision in restorative treatments, particularly in the placement of margins, and of rigorous post‐treatment maintenance protocols. A multidisciplinary approach involving dentists and periodontists is essential for mitigating risks, improving patient outcomes and translating research findings into optimised care strategies.

## 5 Conclusions

The results of the SHIP‐TREND study highlight the significant impact of dental restorations on periodontal health, with crowns demonstrating the most pronounced effects. This aligns with previous evidence that restoration margins, especially when placed subgingivally, can promote biofilm accumulation, trigger inflammation and impair periodontal stability. Notably, signs of periodontal deterioration were already evident shortly after restoration placement.

Our findings support an association in which restorations are linked to poorer periodontal health. However, the association between restoration status and periodontal outcomes at sites adjacent to surfaces with restorations was less pronounced in SHIP‐TREND than in earlier studies. As the quality and technical characteristics of the restorations (e.g., margin adaptation, overhangs and subgingival placement) were not evaluated, the underlying mechanisms remain unclear. Future studies should therefore include detailed assessments of restoration quality and materials to clarify the influence of specific restorative factors on periodontal outcomes.

## Author Contributions

T.K. and B.H. substantially contributed to the conception or design of the work. P.N., T.K., C.P., S.‐E.B., S.R., S.S., S.N., H.V., P.K. and B.H. contributed to the acquisition, analysis or interpretation of data. P.N., T.K. and B.H. drafted the work. C.P., S.S., S.‐E.B., S.R., S.N., H.V. and P.K. revised the draft critically for important intellectual content. All authors approved the final version of the manuscript and are accountable for all aspects of the work.

## Funding

SHIP is part of the Community Medicine Research Network of the University Medicine Greifswald, which is supported by the German Federal State of Mecklenburg‐West Pomerania. C.P. was funded by the Deutsche Forschungsgemeinschaft (DFG, German Research Foundation) – 451892213.

## Ethics Statement

SHIP‐TREND was positively evaluated by the ethics committee of the University of Greifswald (SHIP‐TREND‐0: BB 39/08a; SHIP‐TREND‐1: BB 174/15). All participants were informed about the study protocol and signed the informed consent and the privacy statement.

## Conflicts of Interest

The authors declare no conflicts of interest.

## Supporting information


**Data S1:** jcpe70082‐sup‐0001‐Online_Appendix.docx.

## Data Availability

The data that support the findings of this study are available from Forschungsverbund Community Medicine. Restrictions apply to the availability of these data, which were used under license for this study. Data are available from https://transfer.ship‐med.uni‐greifswald.de/FAIRequest/login with the permission of Forschungsverbund Community Medicine.
