# Supplementary material for: Associations Between Restoration Margins and Adjacent Periodontal Status—Longitudinal Results From SHIP‐TREND
Source: J Clin Periodontol. 2025 Dec 22;53(4):520–8. doi: 10.1111/jcpe.70082 (PMC12972603; doi:10.1111/jcpe.70082)
Supplement: Supplementary file 1 — Data S1: jcpe70082‐sup‐0001‐Online_Appendix.docx. [file JCPE-53-520-s001.docx]

**Supporting Information**

**Associations between restoration margins and adjacent periodontal status – Longitudinal results from SHIP-TREND**

Patrick Nafz, Thomas Kocher, Christiane Pink, Sebastian-Edgar Baumeister, Stefan Reckelkamm, Stefanie Samietz, Sonya Ceesay, Henry Völzke, Philipp Kanzow, Birte Holtfreter

**Materials & Methods**

1. The recommendations of the Strengthening the Reporting of Observational Studies in Epidemiology (STROBE) guidelines for observational studies were applied for reporting﻿ (von Elm et al., 2014).

***Caries examinations***

All examinations were conducted in an illuminated dental chair and with the option to use aspiration or an air jet. Magnification glasses were not allowed. Coronal caries was diagnosed visually using a periodontal probe (PCPUNC 15, Hu-Friedy, Chicago, IL, USA) to touch the tooth surface softly. Coronal caries was examined excluding third molars on a surface level (occlusal, distal, buccal, mesial, palatinal/lingual) and surfaces were classified as sound (excluding persisting teeth of the first dentition), primary caries (including dentine caries only and excluding enamel defects), filled (excluding crowned front teeth after trauma), filled with secondary caries (secondary caries lesions develop exclusively in association with restorations), missing (excluding front teeth extracted after trauma, symmetric extractions of premolars due to orthodontic treatments) in a half-mouth design (randomly chosen left or right side; same side at baseline and follow-up). Information on whether teeth/surfaces were crowned (full or partial crowns; including telescopic crowns, bridges, and similar prosthetic restorations) was obtained from the dental status assessments. Findings from the occlusal surfaces were not considered, as these surfaces are not adjacent to the gingival margin.

***Periodontal examination***

Probing depth (PD) and CAL were measured at distobuccal, midbuccal, mesiobuccal, and midlingual/midpalatinal sites according to the half-mouth method excluding third molars (left or right side randomly selected) using a manual periodontal probe (PCPUNC 15, Hu-Friedy, Chicago, IL, USA). Measurements were rounded to the nearest whole millimetre. Digits between 0.1 and 0.4 were rounded down, and digits between 0.5 and 0.9 were rounded up. PD was measured as the distance between free gingival margin (FGM) and pocket base. If the cemento–enamel junction (CEJ) was located sub-gingivally, CAL was calculated as PD minus the distance between FGM and CEJ. If recession was present at the examined site, CAL was directly measured as the distance between CEJ and the pocket base. Where the determination of the CEJ was indistinct (wedge-shaped defects, fillings, and full crown margins), CAL was not recorded. BOP was recorded at the identical four sites on the first incisor, the canine and the first molar in each probed quadrant. If teeth were missing, the next distally located tooth was assessed. While BOP and PD were assessed at all sites adjacent to surfaces with fillings or crowns (full and partial), CAL was only measured at sites adjacent to sound surfaces or surfaces with fillings or crowns (full or partial) when the CEJ could be identified.

#### **Reliability data**

1. In SHIP-TREND-0, dental examinations were conducted by six calibrated examiners. In calibration exercises, all dentists repeatedly examined five persons not connected to the study. Intra-rater correlations for CAL measurements ranged between 0.67 and 0.89 and inter-rater correlation was 0.70. For PD measurements, the examiners yielded intra-rater correlations between 0.68 and 0.88 and an inter-rater correlation of 0.72. For coronal caries examinations, Cohen’s kappa reliability coefficients were 0.83-1.00 (intra-examiner) and 0.72-1.00 (pairwise inter-examiner). For assessment of the tooth status, Cohen’s kappa reliability coefficients were 0.93-0.99 (intra-examiner) and 0.94-0.98 (pairwise inter-examiner).
2. In SHIP-TREND-1, dental examinations were conducted by six calibrated examiners. In calibration exercises, all dentists repeatedly examined five persons not connected to the study. Intra-rater correlations for CAL measurements ranged between 0.90 and 0.96 and pairwise inter-rater correlations of 0.86-0.94. For PD measurements, the examiners yielded intra-rater correlations between 0.77 and 0.91 and pairwise inter-rater correlations of 0.63-0.85. For coronal caries examinations, Cohen’s kappa reliability coefficients were 0.93-1.00 (intra-examiner) and 0.84-0.98 (pairwise inter-examiner). For assessment of the tooth status, Cohen’s kappa reliability coefficients were 0.97-1.00 (intra-examiner) and 0.91-0.96 (pairwise inter-examiner).

***Laboratory measurements***

Fasting blood samples were drawn from the cubital vein in the supine position and aliquots were prepared for immediate analysis and for storage at −80 °C. HbA1c concentrations were determined by high-performance liquid chromatography (Bio-Rad Diamant, Munich, Germany).

#### **Covariates**

1. Based on the dental interview, toothbrush usage was categorized into four groups: no toothbrush use, manual tooth brush (MTB) use, both MTB and powered toothbrush (PTB) use and PTB use. Toothbrushing frequency was categorized as <2 times daily versus ≥2 times daily. Self-reported periodontal treatment within the last 5 years, regular professional tooth cleaning (≥1 times/year), and dental visits within the last 12 months were recorded. From the computer-assisted personal interview, school education (<10, 10, >10 years), smoking status (never, former, and current smoking) and the food frequency score pattern (FFS; determined according to (Luedemann et al., 2002; Winkler et al., 1995) and categorized as unfavourable, intermediate or recommendable) were retrieved. Equivalence house income was determined as the quotient of the mean value of the respective category of net income and the square root of the number of persons living in the household (Kawachi et al., 1997). Standardized measurements of body height and weight were performed with calibrated scales and the body mass index (BMI) was calculated as weight divided by height squared (kg/m²). Known diabetes mellitus was defined as physician's diagnosis or antidiabetic medication intake (Anatomic Therapeutic Chemical classification system; code A10).

**References**

Kawachi, I., et al. (1997). The relationship of income inequality to mortality: does the choice of indicator matter? *Soc Sci Med, 45*(7), 1121-1127. doi:10.1016/s0277-9536(97)00044-0

Luedemann, J., et al. (2002). Association between behavior-dependent cardiovascular risk factors and asymptomatic carotid atherosclerosis in a general population. *Stroke, 33*(12), 2929-2935. doi:10.1161/01.str.0000038422.57919.7f

von Elm, E., et al. (2014). The Strengthening the Reporting of Observational Studies in Epidemiology (STROBE) Statement: guidelines for reporting observational studies. *Int J Surg, 12*(12), 1495-1499. doi:10.1016/j.ijsu.2014.07.013

Winkler, G., et al. (1995). Kurzmethoden zur Charakterisierung des Ernährungsmusters: Einsatz und Auswertung eines Food-Frequency-Fragebogens. *Ernährungsumschau, 42*, 289-291.

**Table S1.** Adjusted effects estimates from inverse probability weighted generalised estimating equations with cluster-robust standard errors (BOP and PD ≥4 mm: modified Poisson regression; PD, CAL: gamma distribution and log link; with robust standard errors and inverse probability weighting) obtained by regressing the periodontal status at follow-up in Model A on baseline caries status (both from partial-mouth examinations) and in Model B on the change in caries status between baseline and 7-year follow-up (incident fillings and crowns). For Model B we included only sites that were sound and caries-free at baseline. All models were additionally adjusted for the household equivalence income.

|  | **Outcome** | | | |
| --- | --- | --- | --- | --- |
|  | **Having bleeding on probing** | **Probing depth** | **Having a probing depth of ≥4 mm** | **Clinical**  **attachment level** |
| **Model A** | N=43,445 | N=92,324 | N=92,324 | N=74,652 |
| **Number (%) of surfaces being…** |  |  |  |  |
| Sound | 30,412 (70.0%) | 66,524 (72.05%) | 66,524 (72.05%) | 63,661 (85.3%) |
| Filled | 5,922 (13.6%) | 12,349 (13.4%) | 12,349 (13.4%) | 10,697 (14.3%) |
| Crowned | 7,111 (16.4%) | 13,451 (14.6%) | 13,451 (14.6%) | 294 (0.4%) |
| **Effect estimates (with 95% CIs)** | RR with 95% CI | Exp(β) with 95% CI | RR with 95% CI | Exp(β) with 95% CI |
| Sound | Ref. (1.00) | Ref. (1.00) | Ref. (1.00) | Ref. (1.00) |
| Filled | 1.17 (1.10, 1.24) | 1.03 (1.02, 1.04) | 1.18 (1.11, 1.24) | 1.04 (1.03, 1.06) |
| Crowned | 1.42 (1.33, 1.51) | 1.10 (1.09, 1.12) | 1.47 (1.38, 1.57) | 1.20 (1.11, 1.29) |
| **Adjusted percentages/averages (with 95% CIs)** |  |  |  |  |
| Sound | 15.5 (12.9, 18.0) | 1.93 (1.87, 1.99) | 4.6 (3.6, 5.6) | 1.69 (1.58, 1.81) |
| Filled | 18.1 (15.1, 21.1) | 1.99 (1.92, 2.05) | 5.4 (4.2, 6.6) | 1.76 (1.64, 1.88) |
| Crowned | 21.9 (18.2, 25.5) | 2.13 (2.06, 2.20) | 6.8 (5.2, 8.3) | 2.03 (1.82, 2.24) |
| **Model B** | N=30,342 | N=66,385 | N=66,385 | N=63,577 |
| **Number (%) of surfaces being…** |  |  |  |  |
| Sound | 28,078 (92.5%) | 61,491 (92.6%) | 61,491 (92.6%) | 60,675 (95.4%) |
| Incidentally filled | 1,399 (4.6%) | 2,890 (4.4%) | 2,890 (4.4%) | 2,814 (4.4%) |
| Incidentally crowned | 865 (2.9%) | 2,004 (3.0%) | 2,004 (3.0%) | 88 (0.1%) |
| **Effect estimates (with 95% CIs)** | RR with 95% CI | Exp(β) with 95% CI | RR with 95% CI | Exp(β) with 95% CI |
| Sound | Ref. (1.00) | Ref. (1.00) | Ref. (1.00) | Ref. (1.00) |
| Incidentally filled | 1.23 (1.10, 1.36) | 1.03 (1.02, 1.05) | 1.14 (1.03, 1.26) | 1.02 (1.002, 1.04) |
| Incidentally crowned | 1.38 (1.20, 1.58) | 1.10 (1.07, 1.12) | 1.28 (1.10, 1.49) | 1.15 (1.03, 1.28) |
| **Adjusted percentages/averages (with 95% CIs)** |  |  |  |  |
| Sound | 15.6 (12.6, 18.6) | 1.90 (1.84, 1.97) | 3.8 (2.8, 4.7) | 1.71 (1.59, 1.83) |
| Incidentally filled | 19.1 (15.1, 23.2) | 1.97 (1.90, 2.04) | 4.3 (3.1, 5.5) | 1.75 (1.62, 1.87) |
| Incidentally crowned | 21.5 (16.7, 26.4) | 2.09 (2.01, 2.17) | 4.8 (3.4, 6.2) | 1.96 (1.72, 2.21) |

*Note:* Models were adjusted for baseline levels of the outcome variable, probing site, age (cont.), sex, education, smoking, known or diagnosed diabetes, body mass index (cont.), dental visit within last 12 months, food frequency score (cont.), and household equivalence income. The following settings were fixed for the calculation of marginal means: baseline BOP=no; baseline PD=2, baseline PD≥4mm=no, baseline CAL=2, age=50, sex=female, school education=10 years, smoking=never, diabetes mellitus=no, BMI=27, dental visits=no, probing site=midoral, FFS=14 and household equivalence income=1500. BOP was only measured at the first incisors, canines and first molars. Abbreviations: β, beta coefficient; CI, confidence interval; RR, Risk Ratio.

**Table S2.** Effect modification by measurement site/surface: Adjusted effects estimates from inverse probability weighted generalised estimating equations with cluster-robust standard errors (BOP and PD ≥4 mm: modified Poisson regression; PD and CAL: gamma distribution and log link; all models with robust standard errors and inverse probability weighting) obtained by regressing the periodontal status at follow-up in Model A on baseline caries status and in Model B on the change in caries status between baseline and 7-year follow-up (evaluating incident fillings and crowns). For Model B we included only sites that were healthy and caries-free at baseline. Analyses were conducted separately for proximal and non-proximal surfaces/sites.

|  | **Outcome** | | | |
| --- | --- | --- | --- | --- |
|  | **Having bleeding on probing** | **Probing depth** | **Having a probing depth of ≥4 mm** | **Clinical**  **attachment level** |
| **Model A** | | | | |
| **P value for multiplicative interaction term** | 0.001 | <0.001 | <0.001 | <0.001 |
| **Proximal surfaces/sites** | N=22,448 | N=47,685 | N=47,685 | N=38,757 |
| **Number (%) of surfaces being…** |  |  |  |  |
| Sound | 15,242 (67.9%) | 32,341 (67.8%) | 32,341 (67.8%) | 31,315 (80.8%) |
| Filled | 3,517 (15.7%) | 8,372 (17.6%) | 8,372 (17.6%) | 7,283 (18.8%) |
| Crowned | 3,689 (16.4%) | 6,972 (14.6%) | 6,972 (14.6%) | 159 (0.4%) |
| **Effect estimates (with 95% CIs)** | RR with 95% CI | Exp(β) with 95% CI | RR with 95% CI | Exp(β) with 95% CI |
| Sound | Ref. (1.00) | Ref. (1.00) | Ref. (1.00) | Ref. (1.00) |
| Filled | 1.28 (1.20, 1.38) | 1.02 (1.01, 1.03) | 1.17 (1.10, 1.25) | 1.08 (1.06, 1.09) |
| Crowned | 1.44 (1.34, 1.55) | 1.07 (1.06, 1.08) | 1.41 (1.31, 1.51) | 1.19 (1.10, 1.28) |
| **Adjusted percentages/averages (with 95% CIs)** |  |  |  |  |
| Sound | 17.1 (14.1, 20.1) | 2.33 (2.25, 2.41) | 7.7 (6.1, 9.4) | 1.89 (1.77, 2.01) |
| Filled | 22.0 (18.0, 25.9) | 2.37 (2.29, 2.45) | 9.1 (7.1, 11.1) | 2.04 (1.90, 2.17) |
| Crowned | 24.6 (20.3, 29.0) | 2.50 (2.41, 2.58) | 10.9 (8.5, 13.3) | 2.24 (2.01, 2.47) |
| **Non-proximal surfaces/sites** | N=22,453 | N=47,686 | N=47,686 | N=38,278 |
| **Number (%) of surfaces being…** |  |  |  |  |
| Sound | 16,195 (72.1%) | 36,401 (76.3%) | 36,401 (76.3%) | 34,393 (89.8%) |
| Filled | 2,588 (11.5%) | 4,358 (9.1%) | 4,358 (9.1%) | 3,746 (9.8%) |
| Crowned | 3,670 (16.4%) | 6,927 (14.5%) | 6,927 (14.5%) | 139 (0.4%) |
| **Effect estimates (with 95% CIs)** | RR with 95% CI | Exp(β) with 95% CI | RR with 95% CI | Exp(β) with 95% CI |
| Sound | Ref. (1.00) | Ref. (1.00) | Ref. (1.00) | Ref. (1.00) |
| Filled | 1.03 (0.93, 1.13) | 1.05 (1.04, 1.06) | 1.18 (1.01, 1.36) | 1.003 (0.98, 1.03) |
| Crowned | 1.37 (1.26, 1.49) | 1.13 (1.11, 1.14) | 1.64 (1.46, 1.84) | 1.21 (1.10, 1.33) |
| **Adjusted percentages/averages (with 95% CIs)** |  |  |  |  |
| Sound | 14.5 (11.3, 17.7) | 1.90 (1.84, 1.97) | 2.4 (1.7, 3.1) | 1.67 (1.54, 1.79) |
| Filled | 14.9 (11.5, 18.3) | 1.99 (1.93, 2.06) | 2.8 (2.0, 3.7) | 1.67 (1.54, 1.80) |
| Crowned | 19.9 (15.4, 24.4) | 2.15 (2.07, 2.22) | 4.0 (2.8, 5.1) | 2.02 (1.77, 2.26) |
| **Model B** | | | | |
| **P value for multiplicative interaction term** | 0.662 | <0.001 | 0.022 | <0.001 |
| **Proximal surfaces/sites** | N=15,205 | N=32,265 | N=32,265 | N=31,263 |
| **Number (%) of surfaces being…** |  |  |  |  |
| Sound | 14,093 (92.7%) | 29,847 (92.5%) | 29,847 (92.5%) | 29,667 (94.9%) |
| Incidentally filled | 712 (4.7%) | 1,593 (4.9%) | 1,593 (4.9%) | 1,570 (5.0%) |
| Incidentally crowned | 400 (2.6%) | 825 (2.6%) | 825 (2.6%) | 26 (0.1%) |
| **Effect estimates (with 95% CIs)** | RR with 95% CI | Exp(β) with 95% CI | RR with 95% CI | Exp(β) with 95% CI |
| Sound | Ref. (1.00) | Ref. (1.00) | Ref. (1.00) | Ref. (1.00) |
| Incidentally filled | 1.32 (1.15, 1.51) | 1.03 (1.01, 1.05) | 1.18 (1.06, 1.32) | 1.07 (1.04, 1.09) |
| Incidentally crowned | 1.48 (1.24, 1.77) | 1.04 (1.01, 1.08) | 1.16 (0.97, 1.39) | 1.06 (0.90, 1.26) |
| **Adjusted percentages/averages (with 95% CIs)** |  |  |  |  |
| Sound | 16.9 (13.4, 20.4) | 2.34 (2.26, 2.43) | 7.0 (5.3, 8.7) | 1.90 (1.77, 2.02) |
| Incidentally filled | 22.3 (17.0, 27.6) | 2.41 (2.32, 2.51) | 8.2 (6.1, 10.4) | 2.02 (1.88, 2.16) |
| Incidentally crowned | 25.1 (18.6, 31.6) | 2.45 (2.34, 2.56) | 8.1 (5.7, 10.5) | 2.02 (1.66, 2.38) |
| **Non-proximal surfaces/sites** | N=16,155 | N=36,328 | N=36,328 | N=34,355 |
| **Number (%) of surfaces being…** |  |  |  |  |
| Sound | 14,900 (92.2%) | 33,623 (92.55%) | 33,623 (92.55%) | 32,951 (95.9%) |
| Incidentally filled | 731 (4.5%) | 1,396 (3.8%) | 1,396 (3.8%) | 1,340 (3.9%) |
| Incidentally crowned | 524 (3.2%) | 1,309 (3.6%) | 1,309 (3.6%) | 64 (0.2%) |
| **Effect estimates (with 95% CIs)** | RR with 95% CI | Exp(β) with 95% CI | RR with 95% CI | Exp(β) with 95% CI |
| Sound | Ref. (1.00) | Ref. (1.00) | Ref. (1.00) | Ref. (1.00) |
| Incidentally filled | 1.13 (0.96, 1.34) | 1.05 (1.03, 1.06) | 0.99 (0.76, 1.30) | 0.96 (0.93, 0.99) |
| Incidentally crowned | 1.28 (1.07, 1.52) | 1.11 (1.08, 1.14) | 1.37 (1.10, 1.70) | 1.17 (1.02, 1.34) |
| **Adjusted percentages/averages (with 95% CIs)** |  |  |  |  |
| Sound | 13.8 (10.4, 17.3) | 1.88 (1.81, 1.95) | 2.2 (1.4, 2.9) | 1.67 (1.54, 1.80) |
| Incidentally filled | 15.6 (11.1, 20.2) | 1.97 (1.89, 2.05) | 2.1 (1.1, 3.1) | 1.61 (1.48, 1.74) |
| Incidentally crowned | 17.7 (12.6, 22.8) | 2.10 (2.00, 2.19) | 3.0 (1.7, 4.2) | 1.95 (1.64, 2.26) |

*Note:* Models were adjusted for baseline levels of the outcome variable, probing site, age (cont.), sex, education, smoking, known or diagnosed diabetes, body mass index (cont.), dental visit within last 12 months, and food frequency score (cont.). The following settings were fixed for the calculation of marginal means: baseline BOP=no; baseline PD=2, baseline PD≥4mm=no, baseline CAL=2, age=50, sex=female, school education=10 years, smoking=never, diabetes mellitus=no, BMI=27, dental visits=no and FFS=14; for models including only proximal surfaces/sites, probing site was set to ‘distal’; for models including only non-proximal surfaces/sites, probing site was set to ‘midoral’. BOP was only measured at the first incisors, canines and first molars. Abbreviations: β, beta coefficient; CI, confidence interval; RR, Risk Ratio.

**Table S3.** Incidence rates for extraction according to worst caries status (considering oral, buccal, distal, mesial and occlusal surfaces) on tooth level (N=25,714 teeth in 2,276 subjects with baseline caries data and follow-up information on tooth loss).

| Baselines status | Person-time (years) | Number of extractions | Incidence rate per 1000 person-years (95% CI) |
| --- | --- | --- | --- |
| Sound | 99,891.4 | 281 | 2.8 (2.5, 3.2) |
| Filled | 55,859.6 | 415 | 7.4 (6.7, 8.2) |
| Crowned | 28,200.2 | 498 | 17.7 (16.2, 19.3) |

*Note:* For extracted teeth, the event time was set at half the difference between the baseline and the follow-up. Abbreviations: CI, confidence interval.


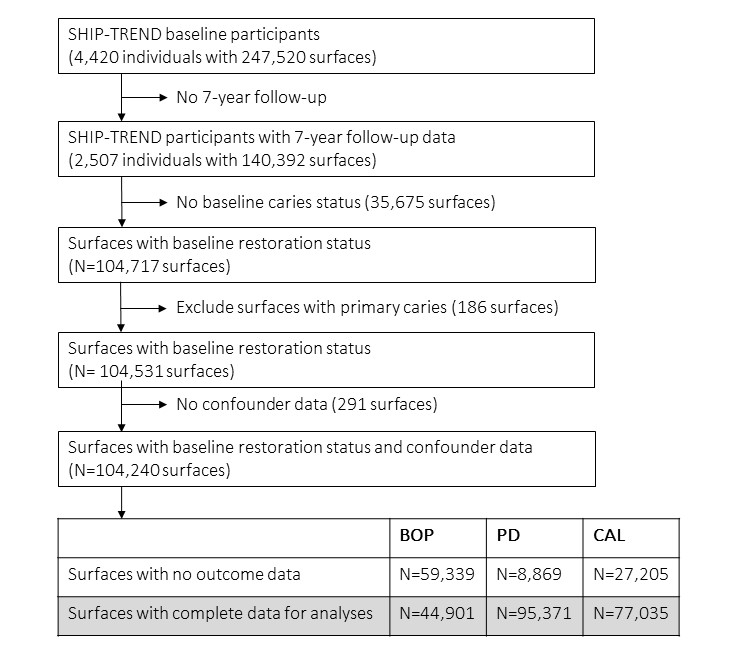


Figure S1. Selection of individuals and surfaces for analysis.
